# Periodization and Programming for Individual 400 m Medley Swimmers

**DOI:** 10.3390/ijerph18126474

**Published:** 2021-06-15

**Authors:** Francisco Hermosilla, José M. González-Rave, José Antonio Del Castillo, David B. Pyne

**Affiliations:** 1 Sport Training Lab, University of Castilla-La Mancha, 45008 Toledo, Spain; fhermosilla@nebrija.es; 2 Facultad de Ciencias de la Vida y la Naturaleza, Universidad Nebrija, 28248 Madrid, Spain; 3 Catalonian Swimming Federation and High Performance Center, Alcalde Barnils, Av. 3-5, Sant Cugat del Vallès, 08174 Barcelona, Spain; kasti71@gmail.com; 4 Research Institute for Sport and Exercise, Faculty of Health, University of Canberra, Bruce, ACT 261, Australia; David.Pyne@canberra.edu.au

**Keywords:** swimming, individual medley, training, season

## Abstract

Knowledge in the scientific domain of individual medley (IM) swimming training over a competitive season is limited. The purpose of this study was to propose a detailed coaching framework incorporating the key elements of a periodized training regimen for a 400 m IM swimmer. This framework was based on the available coaching and scientific literature and the practical experience and expertise of the collaborating authors. The season has been divided in two or three macrocycles, further divided in three mesocycles each (six or nine mesocycles in total), in alignment with the two or three main competitions in each macrocycle. The principal training contents to develop during the season expressed in blood lactate zones are: aerobic training (~2 mmol·L^−1^), lactate threshold pace (~4 mmol·L^−1^) and VO_2_max (maximum oxygen uptake) (~6 mmol·L^−1^). Strength training should focus on maximum strength, power and speed endurance during the season. Altitude training camps can be placed strategically within the training season to promote physiological adaptation and improvements in performance. A well-constructed technical framework will permit development of training strategies for the 400 m IM swimmer to improve both training and competitive performance.

## 1. Introduction

Swimming competitions are performed in four major strokes (front crawl, backstroke, breaststroke and butterfly). The individual medley events (IM) comprise all four swimming strokes in the following order: butterfly, backstroke, breaststroke and freestyle. The two variants of the IM are the 200 m IM (50 m of each stroke) and the 400 m IM (100 m of each stroke). It is necessary to train all four strokes underpinning the widespread assertion in the high performance swimming community that the IM events are the most complex and challenging on swimming program [1]. Gonjo and Olstad [2] highlight the lack of knowledge on comprehensive guidelines for the preparation of high-level 400 m IM swimmers.

In the IM swimming, the energetic and biomechanics differences between the four strokes yield a variable relative contribution of each stroke to the final performance. However, it is not clear which stroke(s) are more important in the final performance in IM events. In the 400 m IM, breaststroke and freestyle seems to be the most relevant stroke in female swimmers [3,4]. In contrast, for male swimmers, the backstroke and breaststroke appear more important [4,5]. When planning training, coaches need to determine then prescribe the relative proportion of training of each stroke for the 400 m IM event throughout the season. To achieve the best performance in 400 m IM, coaches must ensure that middle-distance front crawl training is a priority given a positive association between that freestyle and IM swimming [6].

To maximize the IM swimmers’ performance, it is important to establish a detailed understanding of the training characteristics of both the 200 m and 400 m IM events for effective planning and monitoring. A coach needs to consider the training volume, frequency and intensity distribution for maximizing physical capacity and performance. Periodization can be defined as the macromanagement of the delineated stages of training process with respect to the time allocated toward various elements [7]. The key aspects that underpin periodization are: (i) determining relevant dates (e.g., main and minor competitions), (ii) determining the sequence of phases for each training cycle and (iii) managing load dynamics with the intent of achieving peak or optimal performance at the critical competitions [8]. There are few studies which have examined the key aspects related to the best performance in 400 m IM events, including the training organization and periodization. Therefore, the aim of this narrative review is to examine the evidence on periodization related to the 400 m IM and identify key elements and best practices for this event. We address key aspects, such as bioenergetics necessary to plan the periodization for 400 m IM and examine traditional periodization following two or three peaks performance, training methods and fitness phases for each training period in accordance with other narrative reviews in individual sports [9]. A narrative review provides a historical account of the development of theory and research on a topic (although the contribution to knowledge will be relatively minor [10]. Here we address theoretical conceptualizations, training constructs and relevant scientific literature, to propose a practical framework for preparing 400 m IM swimmers.

## 2. Literature Search Methodology

Electronic searches of PubMed/MEDLINE, SPORTDiscus, Scopus and Web of Science were conducted. The search terms used were “individual medley swimming”, “middle distance swimming training”, “swimming training periodization” and “swimming periodization”. Relevant review articles were also examined to uncover studies which might have been missed in the primary search. The reference list of selected manuscripts was also examined for other potentially eligible manuscripts. No limits regarding the year of publication were employed. Studies were included when (a) they were published in English language (b); provided training zones, volumes and/or periodization details about middle distance or IM events and (c) focused on swimming performance in IM events. Exclusion criteria were: (a) swimmers with a current injury or disability and other aquatic participants (e.g., water polo, diving, triathletes) and (b) studies focusing on pacing or performance trends.

**Figure 1:**
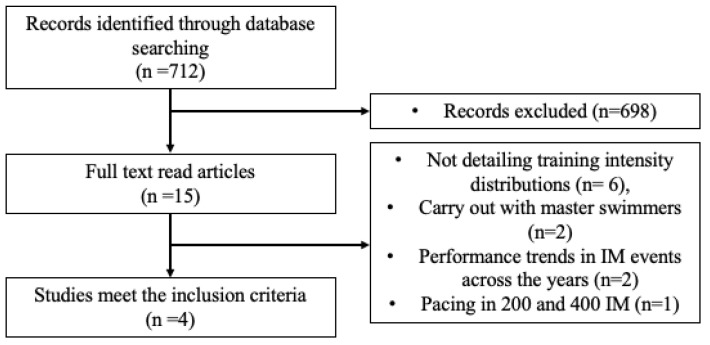
Flow chart summary of the study selection process.

The initial database search identified 714 records that were relevant to the search keywords. After removal of duplicates and elimination of papers based on title and abstract screening, 15 manuscripts remained. Finally, four articles were included in this review [11,12,13,14]. The 11 studies that did not match the eligibility criteria based on full-text screening were discarded for one or more of the following reasons: not detailing training intensity distributions (n = 6), conducted with master swimmers (n = 2), performance trends in IM events across the years (n = 1) and pacing in 200 and 400 m IM (n = 1) (Figure 1).

## 3. Bioenergetics of Individual Medley Events

The 400 IM has a duration ranging from 4 to 4.30 min and is considered as a middle distance swimming event [1]. Middle distance events are supported energetically by a combination of phosphate energy; anaerobic glycolysis and aerobic combustion of carbohydrate, fat and protein [15]. Competitive swimmers spend most of their training time improving aerobic endurance, defined as the ability to sustain a high percentage of VO_2_max for a long period, through careful and repeated interval-based training. This type of training is important for performance in events around 4 min such as the 400 m IM [16]. The physiological preparation for a 400 m IM should address the key physiological factors of the maximal aerobic power (rate of adenosine triphosphate resynthesis), capacity (total amount of adenosine triphosphate resynthesis from available fuels) and VO_2_max (maximum oxygen uptake) [1]. The velocity associated with VO_2_max (vVO_2_max) is the single best predictor of middle-distance swimming performance especially in 400 m events [17,18]. From the data provided by 400 m front crawl swimmers [19,20], the estimated velocity achieved during 400 m IM is ~100% of vVO_2_max. At these intensities, the attainment of a VO_2_ steady state is delayed due to the emergence of a supplementary slowly developing component of the VO_2_ response [21]. The VO_2_ fast component is stable at intensities between 95, 100 and 105%; however, the kinetics of the VO_2_ slow component and the corresponding metabolic profiles showed variations between this intensities [19].

Other important physiological factors include the lactate threshold (LT), the ability to sustain a high percentage of VO_2_max during the competition and the energy cost of locomotion [15,18,19,22]. The physiological adaptations should align with the periodization of each swimmers’ training and competition calendar. These physiological adaptations are usually prescribed with specific training sets and sessions in the pool and dryland training. One common approach in elite-level swimming to enhancing physiological and performance adaptations is incorporation of altitude training (either real or simulated to induce hypoxia).

## 4. Training Monitoring

External load monitoring is usually assessed by quantifying the weekly training volume [23]. The training volumes are usually classified into three or five intensities zones [14]. The three training zone model is typically established using swimming velocity and blood lactate concentrations as follows: z1 ≤ 2 mmol·L^−1^; z2 2–4 mmol·L^−1^, and z3 ≥ 4 mmol·L^−1^ [13]. However, [24,25] proposed a modification with z1 ≤ 3 mmol·L^−1^ and z2 between 3–4 mmol·L^−1^. In swimming, the most common model adopted in the sports science literature comprises five zones: z1 ≤ 2 mmol·L^−1^, z2 2–4 mmol·L^−1^, z3 4–6 mmol·L^−1^, z4 6–10 mmol·L^−1^ and z5 < 10 mmol·L^−1^ [12,13,26]. Training zones can be categorized according to the response in blood lactate concentration: Z1; Aerobic low intensity (A1), z2; Aerobic maintenance (A2), z3; lactate threshold (LT), z4; VO_2_max and intensity above VO_2_max as 200 m race pace and z5 maximal swimming speed [26]. These training zones need to be established and then checked periodically during a training season

Monitoring of heart rate also has been used during training sessions to indicate training intensities [27] but is subject to substantial biological and measurement error. Nevertheless, blood lactate measurements are considered more useful in determining the training intensity because they facilitate better monitoring of the effect of training workloads on the muscle [23]. Thus, blood lactate is a good indicator of the muscles’ capacity for an athletic performance which allows coaches to identify the type and extent of physiological disturbance and the degree of adaptation that has taken place over time [23]. An increase in blood lactate for the same training stimulus may, for example, point to increased anaerobic metabolism, and therefore, higher levels of lactate at slower speeds may be indicative of impending overtraining [28]. Nevertheless, the values of blood lactate are associated with a high between-swimmer variability in swimming techniques, with a range from <2 to >5 mmol·L^−1^ at lactate threshold intensity. Front crawl (3.3 mmol·L^−1^) and breaststroke (2.9 mmol·L^−1^) present lower levels of blood lactate at the lactate threshold intensity than butterfly (4.9 mmol·L^−1^) and backstroke (3.9 mmol·L^−1^) [29]. It is recommended to schedule a blood lactate assessment test using a prescribed testing protocol every few weeks [23].

The Rating of Perceived Exertion (RPE) is another commonly used method for assessing the internal training load [30,31,32,33]. Studies have showed moderate to large correlations between the heart rate and blood lactate concentrations [34,35]. Although, RPE is a valid method for assessing the training stress in high-intensity exercises [36,37], it is important to acknowledge that personal perceptions of physical efforts is a very complex interaction of many factors [38]. Therefore, some investigators recommend to complement the RPE with an objective assessment of internal training load such as blood lactate and/or heart rate monitoring [39,40].

## 5. Training Periodization

Periodization is a process that serves as the macromanagement of the training program in the context of the annual plan [7,41]. Various periodized models such as the reverse linear [24,25,42], or block periodization [43], have been established, but the most common periodized model in swimming is the so-called traditional periodization [14]. Over the recent decades, many periodization approaches have evolved including traditional, blocks, and reverse linear periodization, each offering a differing rationale and template for sub-division of the program into sequential elements [14]. Some authors affirm that the traditional model of periodization can take different forms (i.e., reverse) [44]. Reverse linear periodization has been used in combination with a polarized intensity distribution for improving sprint events. However, the small number of relevant studies did not report any differences with the traditional model in 50 m performance, or a modest improvement of 1% in 100 m performance [24,25]. Polarized training is not recommended for middle distance swimmers; 400 m IM swimmers should benefit from specific periods of training that employ a threshold-oriented training intensity distribution [13].

Training periodization involves the coordination of physical training, psychological capacities training and skill acquisition, providing a comprehensive framework for optimal preparation [45]. Periodization of training leads to a progressive enhancement in the critical physiological and biomechanical factors required for swimming competitions [46]. On this basis, a well-planned and effective periodized approach to training should be established, monitored and refined for swimmers to achieve fitness and peak performance at the major competition for the season [47,48]. In the same way, detailed monitoring of performance and training during the season should be a fundamental aspect to maximize training effectiveness and avoid excessive volume, intensity and/or training load which can cause physiological disturbances (e.g., glycogen depletion, neuromuscular fatigue, decrements in red cell volume and hemoglobin), injuries or illness [49].

The traditional pyramidal model is most commonly used for swimmers characterized by a sequential reduction in training volume moving from zone 1, to zones 2 and 3, respectively. The majority (80%) of the volume is conducted in z1 and the remaining 20% in z2 and z3 [50]. Issurin [51] conceptualized macrocycles as a training period which involved a preparatory and competitive period, usually taking several months. A mesocycle is a medium size training cycle consisting of a number of microcycles which usually involved several weeks, while a microcycle is a small size training cycle consisting of a number of days, frequently one week.

The season can been divided into three macrocycles [52], although the majority of studies reported one or two macrocycles including a comprehensive evaluation conducted on 127 elite swimmers and 20 competitive seasons [12]. This option seems to be the most common used by coaches [53]. Our recommendation is to employ two or three macrocycles divided into three mesocycles each (six or nine mesocycles in total), aligning with three main competitions in each macrocycle to establish an annual plan. In many countries, these competitions comprise in order: short course international championships, national championships and main international competition for the calendar. The emergence of the International Swimming League (ISL) in recent years may require more flexible planning and periodization.

### 5.1. Altitude Training

Altitude training camps during a season can be useful in developing aerobic endurance in world-class endurance athletes [54]. Altitude training elicits an increases in erythropoietic response [55] and hemoglobin mass [56,57]. However, there are also hypoxia-induced non-hematological changes, such as mitochondrial gene expression and enhanced muscle buffering capacity [58,59]. Altitude training can account for ~18–25% of annual training volume in some world-class athletes [60] and is typically performed at altitudes of ~1800–2300 m above sea level or higher [54,60,61]. The duration of an altitude training camp depends on many factors; however, between three to four weeks is suggested for middle distance swimmers [59]. Endurance athletes can undertake altitude training to promote specific training goals of the macrocycle [62]. For example, the early-season training camp when training intensity is typically lower can focus on higher training volumes at low-to-moderate intensities and capitalize on the hematological effects of the hypoxic stimulus [62]. Subsequent training camps should progress to a focus on lactate threshold, aerobic power and VO_2_max training later in the macrocycle. However, a period of low-intensity training during the first few days of altitude acclimatization is recommended [63].

Altitude training camps are generally placed before the main competitions. Six to nine weeks prior to the main competition is typical time to undertake altitude training [59]. Nevertheless, other training camps could be carry out in the middle of the macrocycles to emphasize aerobic training contents [59]. One key aspect that coaches and swimmers must consider is the timing of return from altitude prior to competition. The timing of a peak performance following altitude training is likely to be influenced by a combination of altitude acclimatization and de-acclimatization responses, but more importantly are the periodization of and responses to training and tapering conducted at and after altitude [59]. Previous studies reported periods of between three and five weeks (usually three weeks) [64,65] between altitude training camps and the main competition. This period seems to be optimal timing of post-altitude performance peaking, but individualizing training will be important for optimizing the time to compete after an altitude camp [65].

### 5.2. Preparatory and Main Competitions through the Season

In most individual sports, competitive athletes plan to optimize their performance at the main competition no more than two or three times per year [66]. A retrospective study divided the training season in two macrocycles, the first leading to the national selection trials and the second macrocycle leading to the major international competition) [12]. Each macrocycle should include at least two preparatory (minor) competitions before a major competition. Swimmers performance in the main competitions should faster given the effects of the tapering phase [67] and extra motivation at the main competitions [66]. After a 2-week taper period, swimmers can show an improvement of ~3% in the main competition in comparison with the preparatory competitions carried out three to six weeks before [68]. A common strategy adopted by IM coaches in minor competitions is to have swimmers compete in one or more form stroke events (e.g., butterfly, backstroke or breaststroke) to complement the specific IM events. At the major competitions, IM swimmers typically concentrate on their main event (200 m or 400 m IM), but selection of events will depend on qualifications, team selections and coach/swimmer preferences.

### 5.3. Training Intensity Distribution

Training load variables such as volume, frequency and intensity distribution play an important role in maximizing physical capacity and performance [69]. Annual volume of kilometers for a middle-distance swimmer (400 m freestyle) ranged from 2055 to 2600 km [58]. Increasing the training volume is not be the only way of enhancing performance and more objective and specific training sets are required to improve the quality of the swimming training process [18]. Weekly volume and training intensity distribution are used as a reference for determining a swimmers’ training load and prescribing training sets and sessions. Middle distance swimmers show ranges of training volumes between 39,000 and 42,000 m depending on the type of macrocycles used [12]. However, some training plans showed mean training volumes as high as 58,000 m [11]—peak volumes as high as 70,000–80,000 m—have been reported anecdotally for some international IM and distance swimmers. On this basis, both training volume and intensity distribution should be evaluated together for IM swimmers. A retrospective study showed that middle distance swimmers follow a threshold model in which ~40–44% of the training was performed at an intensity of < 2 mmol·L^−1^ (z1), and 44–46% at 2 to ≤ 4 mmol·L^−1^ (z2) and 9–14% at >4 mmol·L^−1^ (z3) [12]. Threshold-oriented intensity distribution (z1 66%, z2 25%, z3 9%) can improve crucial training contents for 400 m IM swimmers at the velocity at 4 mmol·L^−1^ and VO_2_max [13]. In summary, 400 m IM swimmers should benefit from specific periods of training that employ a threshold-oriented intensity distribution. Coaches should also consider that swimmers who train with a threshold intensity distribution might experience additional fatigue induced by the cumulative impacts of threshold and high-intensity training [13]

### 5.4. Macrocycle Distribution

Training cycles should be prescribed according to the principles of individualization and progression [12]. Two or three distinctive peaks of high total load are suggested in the overall training programs of elite swimmers across the year depending on the number of major competitions scheduled for a particular season. Application of suitable wave-like cycles in units such as a two or three week mesocycle is used to promote physiological adaptations and skill acquisition. Swimmers can engage in mono-, bi- or tri-cycled periodized programs depending on the sequencing of important competitions within that year. An annual periodization composed of two to three macrocycles would be appropriate for Olympic and World Championship seasons [14]. For example, three waves of macrocycles could be planned as follows: the first cycle is conducted from September to December, second from December to April and third from April to August. A two waves macrocycle timeline could be planned as follows: the first cycle from September to April and the second from April to August.

The main aim of the first macrocycle is to develop the general physical fitness and foundation work for specific qualities oriented to the event. The goal of second and third macrocycles is to develop the specific and competitive physiological qualities (VO_2_max, race pace) required for the event, building from general to sport-specific qualities required culminating in the taper at the end of the season. In the two-wave macrocycle, the objectives and training contents of the first and second macrocycles are included in a single macrocycle with the same duration as the first and second cycles in the three wave cycle. Moreover, the second macrocycle of the two-wave cycle has the same duration, training contents and objectives that the third cycle in the three-wave cycle.

#### 5.4.1. First Macrocycle

**Figure 2:**
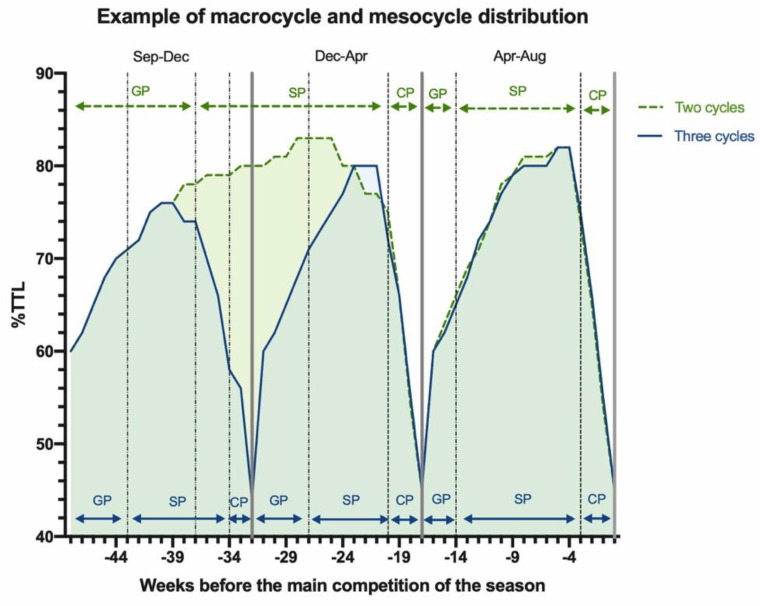
Example of macrocycle and mesocycle distribution. Note: %TTL: Total training load percentage; GP: General Phase; SP: Specific Phase; CP: Competitive Phase.

The beginning of a season in this macrocycle requires development of aerobic endurance up to the lactate threshold, given it is a priority objective on the endurance training for 400 m IM swimmers [53]. The sessions could be performed in short course and long course training depending on the characteristics of the swimmers and the sets planned by coaches. Strength and conditioning (dry-land) training should focus on strength-hypertrophy, maximal strength and strength-metabolic conditioning (e.g., circuit training) with a duration ranging from 50–80 min [70]. Circuit training includes a cardiovascular element in combination with dry-land resistance training using light loads (40–60% one repetition maximum (1 RM)) and brief rest intervals with circuits performed a number of times per session. This work yields metabolic adaptations including an athlete’s buffering capacity [70,71]. Core training sessions can be used to develop stability and postural control of the body position while swimming. Stabilizing muscles can form the basis for generating more strength through the limbs [72]. The competition in this cycle is scheduled in December such as an international short course (for three peaks of performance), but in two peaks of performance, the first cycle is scheduled in April (national selection trial (Figure 2)).

#### 5.4.2. Second Macrocycle

As a general rule, national championships doubling as selection trials are the main competition in this cycle. In the majority of countries, this competition is the qualifying event for the next international competition. The second macrocycle should be characterized by high training volume and high amount of training at z2 (2–4 mmol·L^−1^) and z4 (6–10 mmol·L^−1^) [12]. The objective of these sessions is to improve the aerobic endurance, up to the level of the lactate threshold pace and VO_2_max. In addition, sessions aimed at lactate tolerance and speed could be prescribed as a continuation of the workloads performed in the first macrocycle. The strength and conditioning training should focus on maximal strength, power and speed endurance with resistance exercise. Circuit training is recommended performed in sessions ranging from 30–90 min. During this cycle, the swimmers must continue with the core training sessions for improving their stability. In addition, progressively, the strength training could be transformed from strength-metabolic conditioning to muscle endurance with exercises that simulate the time frame of the event, using light and moderate weights in every exercise (30–50% 1 RM).

#### 5.4.3. Third Macrocycle

The third macrocycle would be the last cycle in the season, featuring the main competition of this cycle which is also the main competition of the season for international swimmers. Clearly, the Olympic Games or the World Championship is the main major competition for achieving the peak performance. The previous competitions should focus on increasing the technical and physical exigency leading to peak performance at the end of the cycle. It is crucial in this cycle to emphasize the technical aspects, especially during the early stages of this cycle, including basic training contents (as aerobic endurance), progressing to specific contents later (as VO_2_max) and, finally, the competitive contents (as race pace). Thus, training in the third macrocycle should be characterized by high amount of training at z2 and z4. Strength and conditioning training is similar to the second macrocycle with a focus on maximal strength, power and speed endurance with resistance exercise. These attributes can be maintained by circuit training to improve aerobic fitness; however, this type of training should be reduced when the main competition approaches.

### 5.5. Mesocycle Distribution

Each cycle is divided in preparatory, competitive and transition following the Matveyev’s proposal [73]. Bompa and Haff [74] reported the preparatory phase has 2 subphases: general phase (GP) and specific phase (SP). The competitive phase (CP) is when the athletes need to peak for a competition. For example, the mesocycle distribution in each macrocycle could keep the following distribution. Three waves macrocycles timeline could be planned as follows, the first cycle: GP: 6 weeks, SP: 10 weeks and CP: 2 weeks; second: GP: 4 weeks, SP: 7 weeks and CP: 3 weeks; and finally the third cycle: GP: 3 weeks, SP: 10 weeks and CP: 3 weeks. Moreover, a two waves macrocycle timeline could be planned as follows, first cycle: GP: 12 weeks, SP: 17 weeks and CP: 3 weeks; second: GP: 3 weeks, SP: 10 weeks and CP: 3 weeks.

#### 5.5.1. General Phase

The main objective of the general phase is to induce physiological, psychological, and technical adaptations that serve as the foundation for competitive performances [75]. The development of aerobic or oxidative endurance should be the main objective in this phase (see Table 1). Lactate threshold and VO_2_max sets could be included in the last few weeks of these mesocycle. Front crawl would be the recommended stroke to perform this type of session because of the large volume required in aerobic sets. However, coaches should consider conducting mixed sets with other strokes. To improve the aerobic endurance, swimmers should train at hart rate 40 to 30 beats below maximum. The suggested pace is half of personal best 200 m time plus 10 to 15 sec. The repeat distances to use when training in this category are 200 to 1500 m [76]. Moreover, lactate threshold and VO_2_max training could be placed in the final weeks of the general mesocycle as introductory sets for the subsequent mesocycle. It would be recommended that a training volume between 55–65 km per week during this phase is appropriate for most 400 m IM swimmers. Dryland training during the general mesocycle is focused on the strength and hypertrophy development. During the first cycle, strength and hypertrophy development are the main objectives, whereas in the second and third cycles coaches should ensure an appropriated maximal strength development.

#### 5.5.2. Specific Phase

As the swimmers progresses to the specific phase of training, it is important maintain the level of physical development established during the preparatory phase. The aerobic endurance training needs to be maintained through this phase. However, the training should be focus on the lactate threshold and VO_2_max development (see Table 2). In the case of the lactate threshold development, swimmers should train at heart rate from 30 to 20 beats below the maximum in repetitions of 50 to 400 m at seven to ten second plus the half of personal best time in 200 m. For 400 m IM swimmers, it is recommended 3000 to 4500 m sets [53]. Finally, VO_2_max sets should be performed in sets of 300 to 500 m with 50 to 150 m repetitions. The suggested pace for VO_2_max training is half of personal best 200 m time plus four to seven seconds [76]. Moreover, during the middle and the last part of this period, the race pace training should be included. Race pace training can be carry out as broken swims (a training repeat with more than one break) and splits (a training repeat with one break) [53]. The specific sets should be performed in a mix of strokes, not only in one stroke, and depending on the week, the training volume should oscillate between 65–90 km per week. During the training first cycle, the dryland training should be focus on maximal strength development; nevertheless, during the second and third training cycles, power and speed endurance development are the main objectives of the dryland training. Maximum strength training could involve exercises as Hamstrings (training machine), Leg press, Dumbbell Row, Chin ups, Lunges Back, Squat, Row Hammer, Bench Press and Triceps overhead (pulley) with one or two sets with an intensity between 70–90% 1 RM and four-six repetitions.

#### 5.5.3. Competitive Phase

Among the main tasks of the competitive phase is perfection of all training factors, which enables the athlete to compete successfully in the main competition or championships targeted by the annual training plan [75]. A primary goal of the competitive and tapering mesocycles is to remove fatigue to stimulate a supercompensation of performance. During the competitive mesocycle, the most important training content is race pace training (see Table 3) and dryland training where the focus on is power (explosiveness) development (see Table 4). The tapering phase prior to international championships can include minor or major competitions, moderate volume including more low intensity training [49,77]. The training volume is progressively decreased for performing a progressive sloped taper phase, with the aim of achieving the best performance in the main competition. An optimal taper duration appears to be between 8–21 days involving a decrease in training load of ~40–60% [75]. Mujika and Padilla [49] recommended a training load reduction between 60–90% and maintaining the training intensity to avoid detraining, provided reductions in the other training variables allow (i.e., fewer training sessions per week or volume) for sufficient recovery to optimize performance. The progressive reduction on the training volume begins around 60 km per week and finishes around 20–30 km per week. The remarkable decrease in volume took place in the second and third macrocycles of the season [77].

## 6. Conclusions

Knowledge of the preparation and periodization of IM training over a season is limited in both the coaching and sports science literature. Progressive development of the critical energetic and biomechanics variables involves the design, implementation and evaluation of an effective IM training plan. The training program we detailed here was organized with a traditional periodization paradigm using two or three macrocycles for the season (with two to three main competitions) incorporating a series of altitude camps. It is incumbent upon the coach to adjust the programming based on individual responses and swimmers’ characteristics to optimize the training process for each swimmer. Future investigations into IM training should determine the long-term effects of individual elite swimmers. It would also be informative to investigate the effects of different periodization models and training loads distribution on the performance in 400 IM using observation analyses of elite swimmers and controlled studies with high-level emerging swimmers.

## Figures and Tables

**Table 1:** Aerobic development and mixed-endurance training sessions and hypertrophy-maximum strength development training set in the general mesocycle (final part) of the first cycle.

Swimming Training									
Objective	Set	Volume (m)		Training	Intensity		Training Zone	Stroke	Notes
Aerobic development and mixed-endurance training	1	2400	3 × (8 × 100)/1:20–1:30–1:40 min	A2, LT, VO_2_max	Z2, Z3, Z4	FC	Performed as training testing, speed control, HR, stroke, frequency and [La^-^]		
	2	3600	36 × 100/1:20–1:30–1:40 min	Sequence: 3 × 100 A2 < 2 × 100 LT < 1 × 100 VO_2_max	Z2, Z3, Z4	FC	3 intensities simultaneously with the [La^-^] of the previous intensity in the next one		
**Strength Training**									
Objective		Cycle	Sets	Exercise		Repetitions		Intensity	
Hypertrophy and maximal strength development		2	4	Short pull Hammer		8		65 < 75%RM	
				Row Hammer		6		80 < 85%RM	
				Pull over		8		70%RM	
			3	Pulls-Up (Eccentric)		3		Body weight	
				Pulls-UP (Supine grip)		3			
			6	Squat		8		85%RM	
				Hamstrings		8		75%RM	

Note: Heart rate (HR); Blood lactate ([La^-^]); Front Crawl (FC); Aerobic maintenance (A2); LT: lactate threshold (LT); Maximum oxygen uptake (VO_2_max); Repetition maximum (RM); Zone 1 (Z2); Zone 2 (Z2); Zone 3 (Z3); Zone 4 (Z4); Zone 5 (Z5). The recommendations made in this table are based on both scientific and empirical data.

**Table 2:** Aerobic and lactate threshold set in the specific mesocycle of the second cycle of the season.

Objective	Cycle	Set	Total Volume (m)	Training	Intensity	Training Zone	Stroke	Notes
Aerobic development and high intensity (lactate threshold) training along with aerobic endurance training	1	1	3600	1 × (300 m/3:45 min)	A1	Z1	FC	Intensity progression in each distance
				1 × (400 m/5 min) + 1 × (500 m/6:30 min)	A2, LT	Z2-Z3		
		2	4000	1 × (400 m/5:15 min) + 8 × 50/50 s	A2, LT	Z2-Z3	BT	
				2 × (200 m) + 4 × 100/1:30 min)	A2, LT	Z2-Z3	BS	
				1 × (400 m/5 min) + 4 × (100 m/1:10 min)	A2, LT	Z2-Z3	FC	
		3	1000	4 × (25 m/1:30 min)	Max	Z5	UUS	
				4 × (50 m/2 min) + 1 × (100/2 min)	400 Race pace	Z4	FC	
				4 × (150 m/2 min)	A2 (last at LT)	Z2, Z3	FC	

Note: Underwater undulatory swimming (UUS); Breaststroke (BK); Front crawl (FC); Butterfly (BT); Backstroke (BS); Aerobic low intensity (A1); Aerobic maintenance (A2); LT: lactate threshold (LT); Zone 1 (Z2); Zone 2 (Z2); Zone 3 (Z3); Zone 4 (Z4); Zone 5 (Z5). The recommendations made in this table are based on both scientific and empirical data.

**Table 3:** Race pace through mixed-endurance training set in the beginning of the competitive mesocycle of the second cycle.

Objective	Cycle	Set	Volume (m)	Training	Intensity	Training Zones	Stroke	Notes
Race pace through mixed-endurance sessions	3	1	2200	2 × (8 × 50 m/1 min) + 1 × 400 m/5:30 min	400 m race pace + A2	Z4-Z2	FC	Combination 400 m speed, focusing on frequency/speed competitive and A2
			1000	4 × (1 × 50 m/1:15 min) + 1 × 200/3 min	200 m race pace + A2/LT	Z4-Z2-Z3	FC	Intensity increase looking for a [La^-^] accumulation
			800	4 × (150 m/2:15 min + 50 m/1 min)	LT + Max speed	Z3-Z5	FC	The aim is to increase the intensity, focusing of AT and 50 m maximal speed
		2	800	8 × (50 m/50 s) + 2 × (200/2:30 min)	200 m race pace + A2	Z4-Z2	FC	The aim is to focus on frequency/competitive 400 m speed
			1000	10 × (100 m/2–2:15 min)	400 m race pace	Z4	1 × BT, 2 × BS, 3 × BK, 4 × FC	The aim is to simulate the competition as much as possible
			400	8 × (50 m/1 min)	Max speed	Z5	FC	The aim is to simulate the last part of the competition
			800	4 × (200 m/2:30 min)	A2	Z2	FC	The aim is to remove the lactate at AT intensity

Note: Blood lactate ([La^-^]); Breaststroke (BK); Front crawl (FC); Butterfly (BT); Backstroke (BS); Aerobic low intensity (A1); Aerobic maintenance (A2); LT: lactate threshold (LT); Maximum oxygen uptake (VO_2_ max_)_; Zone 1 (Z2); Zone 2 (Z2); Zone 3 (Z3); Zone 4 (Z4); Zone 5 (Z5). The recommendations made in this table are based on both scientific and empirical data.

**Table 4:** Power training set in the beginning of the competitive mesocycle of the second cycle.

Objective	Cycle	Sets	Exercise	Repetitions	Intensity
Power development	2	3×	Push-ups (additional weight)	4	105–110% BW
			Bench Press	Max	60% RM-0.9 m/s
		5×	Chin-ups (Eccentric)	4	Body weight
			Row Hammer	Max	60% RM-0.9 m/s
		3×	Isometric Squat	20’’	Body weight
			Squat	Max	60% RM-0.9 m/s

Note: Body weight (BW); Repetition maximum (RM); Meters per second (m/s). The recommendations made in this table are based on both scientific and empirical data.

## Data Availability

Not applicable.
